# Huangzhi Oral Liquid Prevents Arrhythmias by Upregulating Caspase-3 and Apoptosis Network Proteins in Myocardial Ischemia-Reperfusion Injury in Rats

**DOI:** 10.1155/2015/518926

**Published:** 2015-05-17

**Authors:** Xu Ran, Jian Xin Diao, Xue Gang Sun, Ming Wang, Hui An, Guo Qiang Huang, Xiao Shan Zhao, Wen Xiao Ma, Feng Hua Zhou, Yun Gao Yang, Can Ming Miao

**Affiliations:** ^1^Zhongshan Hospital of Traditional Chinese Medicine Affiliated to Guangzhou University of TCM, Zhongshan, Guangdong 528400, China; ^2^The Key Laboratory of Molecular Biology, State Administration of Traditional Chinese Medicine, School of Traditional Chinese Medicine, Southern Medical University, Guangzhou 510515, China; ^3^ZhuJiang Hospital of Southern Medical University, Guangdong, Guangzhou 510515, China

## Abstract

To study the effect of Huangzhi oral liquid (HZOL) on I/R after 2 h and 4 h and determine its regulatory function on caspase-3 and protein networks. 70 SD male rats were randomly divided into seven groups and established myocardial I/R injury model by ligating the left anterior descending coronary artery. Myocardial infarction model was defined by TTC staining and color of the heart. The levels of CK-MB, CTnI, C-RPL, SOD, and MDA were tested at 2 h and 4 h after reperfusion. HE staining and ultramicrostructural were used to observe the pathological changes. The apoptotic index (AI) of cardiomyocyte was marked by TUNEL. The expression levels of caspase-3, p53, fas, Bcl-2, and Bax were tested by immunohistochemistry and western blot. HZOL corrected arrhythmia, improved the pathologic abnormalities, decreased CK-MB, CTnI, C-RPL, MDA, AI, caspase-3, p53, fas, and Bax, and increased SOD ans Bcl-2 with different times of myocardial reperfusion; this result was similar to the ISMOC (*P* > 0.05). HZOL could inhibit arrhythmia at 2 and 4 h after I/R and ameliorate cardiac function, which was more significant at 4 h after reperfusion. This result may be related to decreased expression of caspase-3, p53, and fas and increased Bcl-2/Bax ratio.

## 1. Introduction

The function of apoptosis in cardiovascular disease is gaining recognition. Necrosis is a myocardial cell injury caused by myocardial ischemia. Recent studies demonstrated that oxidative stress and ischemia/reperfusion (I/R) injury not only cause myocardial necrosis but also induce cardiomyocyte apoptosis. The degree of myocardial cell apoptosis is associated with the I/R time [1]. Apoptosis is controlled by a series of pathological processes that mediate the signaling pathway; blocking the signaling pathway helps block apoptosis, thereby preventing myocardial apoptosis and improving heart function [2]. Increasing evidence has indicated that traditional Chinese medicine (TCM) significantly influences the protection of myocardial apoptosis [3]. TCM provides alternative options for preventing myocardial apoptosis to ameliorate the prognosis of I/R. Huangzhi prescription is a TCM that has long been used in China. Previous literature indicated that Huangzhi can improve blood viscosity and hyperlipidemia in patients [4]. Huangzhi oral liquid (HZOL) consists of Huangzhi prescription boiled and alcohol sunk. HZOL ameliorates coronary heart disease by promoting blood circulation and removing blood stasis and turbidity for gasification [5]. However, the effects of HZOL on I/R have been rarely explored. In one of our previous prospective animal studies [6], we found that HZOL can significantly improve cardiac and coagulation functions. The mechanism involved in apoptosis has not been reported. To confirm the effects of HZOL on I/R and explore its potential mechanism, this study was performed.

## 2. Materials and Methods

### 2.1. Preparation of HZOL

HZOL consists of leech, rhubarb, and Fructus arctii, which were mixed at a ratio of 5 : 3 : 3, with a concentration of 2.2 g/mL. HZOL was purchased from the Affiliated Zhongshan Hospital of Traditional Chinese Medicine for Guangzhou University of Chinese Medicine (Guangzhou, China). The quality of HZOL was controlled by thin layer chromatography analysis. The solution was stored in aliquots (10 mL/vase) at −20°C.

### 2.2. Preparation of Rat I/R Model

Male Wistar rats (SPF, no. 0099755, 200 g to 240 g) were purchased from the Experimental Animal Center of Southern Medical University, housed individually in clear plastic cages at a temperature- and humidity-controlled environment with a 12 h light/dark cycle (23 ± 1°C; 12 h light/dark cycle, light on at 7 a.m.) and given ad libitum access to rodent chow and water. Animals were handled in accordance with the Guidelines of Animal Care at Southern Medical University.

A total of 70 rats were randomly divided into seven groups, namely, the Model-2 h group (reperfusion for 2 h after 30 min of myocardial ischemia), Model-4 h group (reperfusion for 4 h after 30 min of myocardial ischemia), HZOL-2 h group (application of HZOL before Model-2 h), HZOL-4 h group (application of HZOL before Model-4 h), isosorbide mononitrate capsule- (ISMOC-) 2 h group (application of ISMOC before Model-2 h), ISMOC-4 h group (application of ISMOC before Model-4 h), and sham group (subjected to the same surgical procedure in the absence of left anterior descending (LAD) coronary artery). Rats in the HZOL-2 h, HZOL-4 h, ISMOC-2 h, and ISMOC-4 h groups were intragastrically administered with HZOL (2 g/kg/d) and ISMOC (3.6 mg/kg/d) at 1 h before surgery. This procedure was performed for seven consecutive days. Normal saline was used as control. Rats were anesthetized with pentobarbital sodium (80 mg/kg, Fluka), intubated, and ventilated artificially using a rodent ventilator (SAR-830, IITC, USA). Ischemia-reperfusion made mould method as mentioned in the paper [7]. Coronary artery occlusion was confirmed by epicardial cyanosis, ST-segment elevation, and an increase in R-wave amplitude. Reperfusion was achieved by releasing the snare and confirmed by recovery from cyanosis and reversal of ECG changes [8]. Rats in the sham group were subjected to the same surgical procedure in the absence of LAD coronary artery. We not only observed cyanosis of the left ventricle using ECG, but also performed 30 min of myocardial ischemia in four rats randomly selected from the sham (two rats) and model groups (two rats). After ischemia, the suture around the LAD coronary artery was retightened, and 1 mL of 1% triphenyl tetrazolium chloride (TTC) stain was injected via the thoracic aorta. After 10 min, the heart was cut into five to six transverse slices, which were 2 mm thick and parallel to the atrioventricular groove. The infarct area was confirmed as the unstained part in the risk area following TTC.

### 2.3. Assessment of Arrhythmias

The incidence of arrhythmias was determined and diagnosed based on the criteria of the Lambeth Conventions [9], including ventricular tachycardia (VT), ventricular fibrillation (VF), and other types of arrhythmias (single extrasystoles, bigeminy, and salvos).

An arrhythmia score was used to evaluate the severity of arrhythmias by giving a grade to each animal as follows: 0 = no arrhythmias; 1 = less than 10 s of VT or other arrhythmias, no VF; 2 = 11 s to 30 s of VT or other arrhythmias, no VF; 3 = 31 s to 90 s of VT or other arrhythmias, no VF; 4 = 91 s to 180 s of VT or other arrhythmias, and/or less than 10 s of reversible VF; 5 = more than 180 s of VT or other arrhythmias, and/or more than 10 s of reversible VF; 6 = irreversible VF [10].

### 2.4. Exclusion Criteria

Experiments were terminated or excluded from the final data analysis if any of the following conditions occurred: absence of signs of successful coronary artery occlusion, severe arrhythmias prior to LAD occlusion and reperfusion, or severe atrioventricular block during the first 5 min of ischemia [11]. Four rats were unsuccessful (Model-2 h, Model-4 h,* *HZOL-2 h, and ISMOC-4 h groups).

### 2.5. Sample Preparation after Reperfusion

5 mL of blood was collected through the abdominal aorta of live rats to detect levels of serum creatine kinase mb isoenzyme (CK-MB), cardiac troponin I (CTnI), C reactive protein (CRP), superoxide dismutase (SOD), and malondialdehyde (MDA). The AAR of the left ventricle of rats in the sham, Model-2 h, Model-4 h, HZOL-2 h, HZOL-4 h, ISMOC-2 h, and ISMOC-4 h groups was removed and divided into four parts. One part was fixed in 10% formalin, another was fixed in 0.1 M phosphate buffer containing 2.5% glutaraldehyde and 2% paraformaldehyde, and the other two parts were flash-frozen in liquid nitrogen and stored at −80°C for use.

### 2.6. Serum Examination

Serum CK-MB, CTnI, CRP, SOD, and MDA determination. The serum biochemical parameters CK-MB, CTnI, and CRP, which closely reflect cardiac function [12], were analyzed by the clinical laboratory of Nanfang Hospital Affiliated to Southern Medical University (Guangzhou, China). The serum levels of SOD and MDA were determined using an SOD and MDA detection kit (Jiancheng, Nanjing, China). The assay was performed according to the manufacturer's instructions.

### 2.7. Histological and Transmission

Electron microscopy (TEM) examination part of the AAR of the left ventricle was fixed in 10% formalin, embedded in paraffin, cut into 4 *μ*m sections, and mounted on slides. The samples were stained with hematoxylin and eosin (HE) for histopathological examination. For TEM examination, samples containing a 2 mm portion from the edge of the incision were immediately fixed in 0.1 M phosphate buffer containing 2.5% glutaraldehyde and 2% paraformaldehyde for 4 h. The samples were then fixed with 1% osmium tetroxide for 2 h, dehydrated through a graded ethanol series, and embedded in epoxy resin. Resin-embedded blocks were cut into 60 nm to 80 nm ultrathin sections with an ultramicrotome (PT-XL, RMC, USA). The ultrathin sections were placed on carbon-coated nickel grids and examined with an H-7500 transmission electron microscope (H-7500, Tokyo, Japan) operating at 80 kV.

### 2.8. Apoptosis Assay

We purchased a terminal deoxynucleotidyl transferase dUTP nick end labeling (TUNEL) apoptosis assay kit for paraffin sections from Nanjing KeyGen Biotech. Inc. (Nanjing, Jiangsu, China). Based on the manufacturer's instructions, all the procedures were performed. Cells were defined as apoptotic if the entire nuclear area of the cell was positively labeled. The apoptotic cells and bodies were counted in five high-power fields. The apoptotic index (AI) was calculated as the percentage of positively stained cells using the following equation: AI = number of apoptotic cells/total number of nucleated cells [13].

### 2.9. Immunohistochemistry

Immunohistochemical staining for caspase-3, p-53, fas, Bcl-2, and Bax was performed using routine immunohistochemistry streptavidin peroxidase method. This method contained a rabbit polyclonal IgG antibody against caspase-3 (1 : 100; Cell Signaling Technology Inc., no. 9662, USA), p-53 (1 : 50; Bioworld Technology Inc., BS3736, Louis Park, USA), fas (1 : 100; Assay Designs, ADI-AAP-221D, USA), Bcl-2 (1 : 50; Bioworld Technology Inc., BS1511, Louis Park, USA) (Cell Signaling Technology, no. 2876, USA), and Bax (1 : 50; Bioworld Technology Inc., BS2538, Louis Park, USA) (Cell Signaling Technology, no. 2772, USA). Nuclear counterstaining was performed using hematoxylin. Five randomly selected fields from each section were examined at a magnification of ×200 and analyzed using Image-Pro Plus 6.0. The positive content (PC) was calculated using the following formula: PC = mean optical density × positive area [10].

### 2.10. Western Blot

Aliquots of heart tissue (50 mg) were homogenized in liquid nitrogen and dissolved in lysis buffer. Protein concentrations were determined by BCA protein quantitative assay. The protein lysates were loaded onto 10% SDS-polyacrylamide gel for separation, electrotransferred to PVDF membranes, and blocked in 5% nonfat milk in Tris-buffered saline, Membranes were incubated overnight using primary antibodies, (caspase-3 (Cell Signaling Technology Inc., no. 9662, USA) diluted to 1 : 1000, p-53 (Bioworld Technology Inc., BS3736, Louis Park, USA) diluted to 1 : 500, fas (Assay Designs, ADI-AAP-221D, USA) diluted to 1 : 500, Bcl-2 (Cell Signaling Technology, no. 2876, USA) diluted to 1 : 1000, and Bax (Cell Signaling Technology, no. 2772, USA) diluted to 1 : 1000) at 4°C. This step was followed by secondary antibodies, which were conjugated using horseradish peroxidase. We performed enhanced chemiluminescence (Merck-Millipore, Germany) detection. The images were captured and documented using a CCD system (image station 2000MM, Kodak, Rochester, NY, USA). Quantitative analysis of these images was performed using Molecular Imaging Software Version 4.0, which was provided by Kodak 2000MM System. The optical density was normalized against actin [10].

### 2.11. Statistical Analysis

Each experiment was repeated at least three times. Data were represented in the form of means ± SD. Data were analyzed using SPSS statistical package (version 13.0, Armonk, NY, USA). Mean values were compared using one-way ANOVA, and multiple comparisons were performed. Data were analyzed using a homogeneity test for variance. If the variances were homogeneous, mean values were compared through ANOVA. The differences between two groups were analyzed based on least significant difference test. If the variances were not homogeneous, mean values were compared using Welch's test. The differences between two groups were analyzed by Games-Howell. Statistical significance was set at *P* < 0.05.

## 3. Results

### 3.1. Effects of HZOL for I/R 2 h- and I/R 4 h-Induced Arrhythmias and Infarct Size

**Figure 1:**
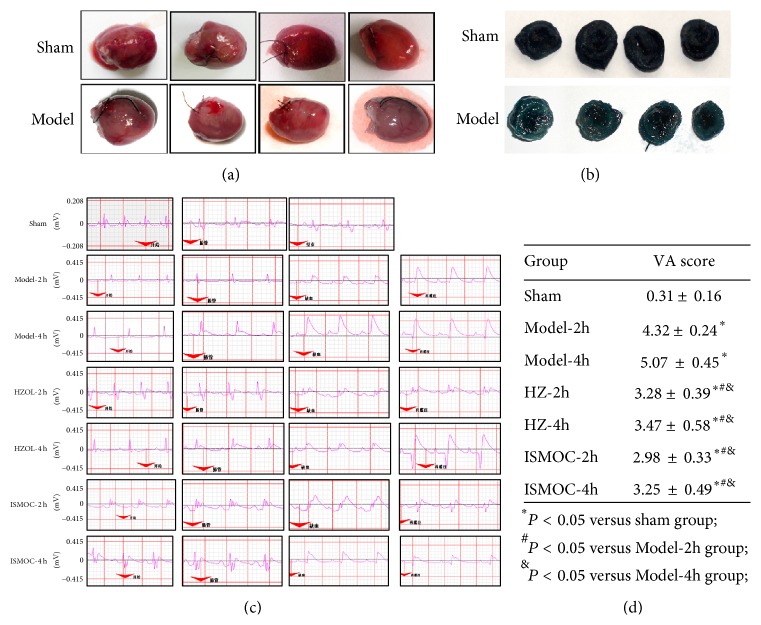
Histological and electrocardiography (ECG) parameters. (a) Representative gross images of whole hearts. (b) Representative of 1% triphenyl tetrazolium chloride (TTC) staining results. (c) Representative of 2-lead electrocardiogram results. (d) Arrhythmia was scored according to 2-lead electrocardiogram. Model-2 h group (after 30 min of myocardial ischemia, reperfusion 2 h), Model-4 h group (reperfusion for 2 h after 30 min of myocardial ischemia), Model-4 h group (reperfusion for 4 h after 30 min of myocardial ischemia), HZOL-2 h group (application of HZOL before Model-2 h), HZOL-4 h group (application of HZOL before Model-4 h), isosorbide mononitrate capsule (ISMOC)-2 h group (application of ISMOC before Model-2 h), ISMOC-4 h group (application of ISMOC before Model-4 h), and sham group (subjected to the same surgical procedure in the absence of left anterior descending coronary artery). ^#^ *P* < 0.05 versus sham group; ^&^ *P* < 0.05 versus Model-2 h group; ^∗^ *P* < 0.05 versus Model-4 h group.

Naked eye observations showed that the heart color from the model group was paler than that of the sham group (Figure 1(a)). Effects of different treatments on I/R-induced arrhythmias are shown in Figure 1(c). In the sham group, neither VT nor VF was observed, and only a few ventricular premature beats appeared during the entire procedure. By contrast, almost all rats in the Model-2 h and Model-4 h groups experienced obvious ST-segment elevation, VT occurrence, and high VF frequency. Furthermore, the frequency of arrhythmias was significantly reduced in the Model-2 h and Model-4 h groups. During reperfusion, malignant arrhythmias were observed in the rats of the I/R group. Pretreatment with HZOL and ISMOC markedly reduced the mean duration of VT (*P* < 0.05), VF (*P* < 0.05), and arrhythmia score compared with those of the I/R group (Figure 1(d)).

### 3.2. Effects of HZOL for I/R 2 h and I/R 4 h on the Serum Levels of CK-MB, CTnI, CRP, SOD, and MDA

**Figure 2:**
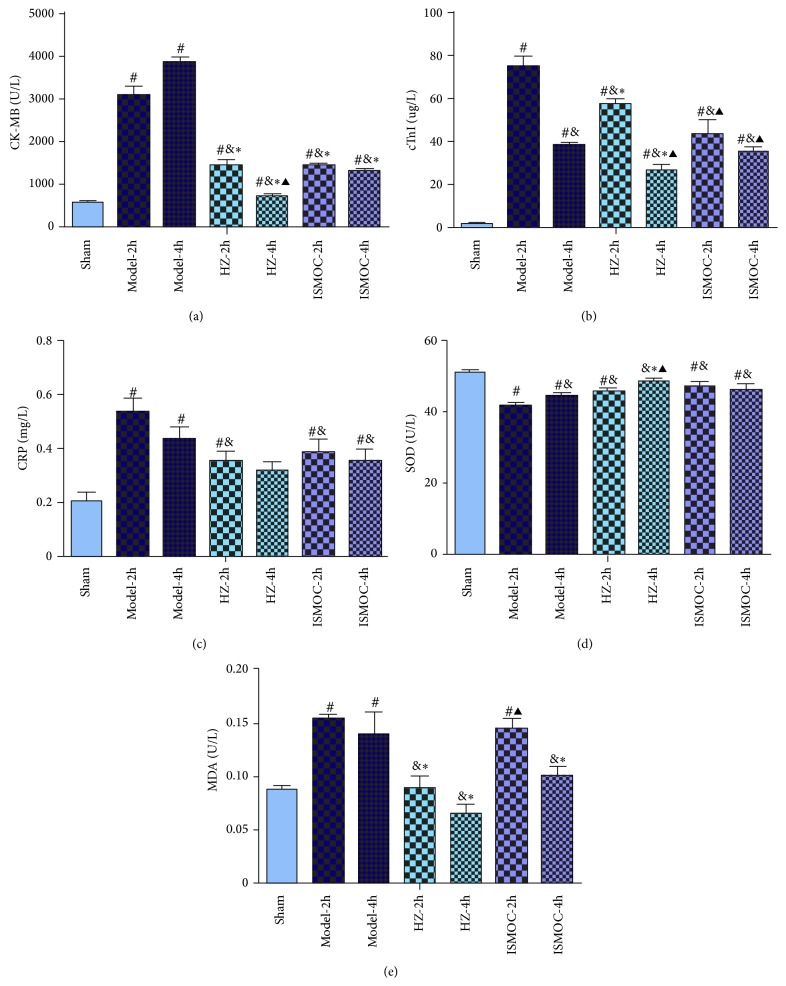
The serum levels of CK-MB, CTnI, CRP, SOD, and MDA after treatment. (a) CK-MB; (b) CTnI; (c) CRP; (d) SOD; (e) MDA; Model-2 h group (after 30 min of myocardial ischemia, reperfusion 2 h), Model-4 h group (reperfusion for 2 h after 30 min of myocardial ischemia), Model-4 h group (reperfusion for 4 h after 30 min of myocardial ischemia), HZOL-2 h group (application of HZOL before Model-2 h), HZOL-4 h group (application of HZOL before Model-4 h), isosorbide mononitrate capsule (ISMOC)-2 h group (application of ISMOC before Model-2 h), ISMOC-4 h group (application of ISMOC before Model-4 h), and sham group (subjected to the same surgical procedure in the absence of left anterior descending coronary artery). ^#^ *P* < 0.05 versus sham group; ^&^ *P* < 0.05 versus Model-2 h group; ^∗^ *P* < 0.05 versus Model-4 h group; ^▲^ *P* < 0.05 versus HZOL-2 h group.

The serum levels of CK-MB, CTnI, CRP, SOD, and MDA after treatment are shown in Figure 2. Results show that CK-MB, CTnI, CRP, and MDA significantly increased, whereas SOD significantly decreased after Model-2 h and Model-4 h (*P* < 0.05). All these parameters indicate severe myocardial ischemia. However, the HZOL-2 h, ISMOC-2 h, HZOL-4 h, and ISMOC-4 h groups could improve cardiac function by decreasing CK-MB, CTnI, CRP, and MDA and increasing SOD levels (*P* < 0.01). The effects were more significant in the HZOL-4 h and ISMOC-4 h groups. Compared with the HZOL-2 h group, the HZOL-4 h group showed more significant effects by decreasing CK-MB and CTnI and increasing SOD levels (*P* < 0.05).

### 3.3. Effects of HZOL for I/R 2 h and I/R 4 h on the Histological and Ultrastructural Changes in the Myocardium

**Figure 3:**
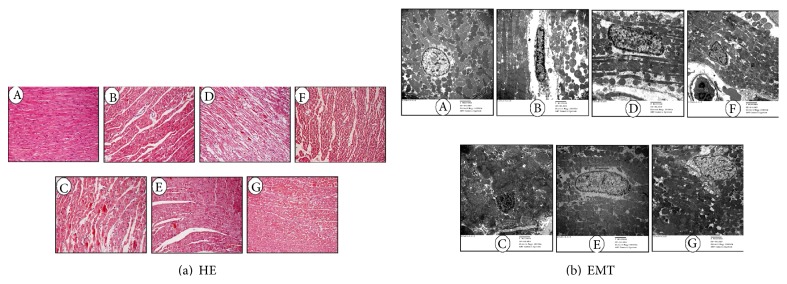
The effects of HZOL for I/R 2 h and I/R 4 h on the histological and ultrastructural changes in the myocardium (a) representative microscopic images of HE stain. (b) TEM images of ultrathin sections of myocardial tissue are changed. A: sham group; B: Model-2 h group; C: Model-4 h group; D: HZOL-2 h group; E: HZOL-4 h group; F: ISMOC-2 h group; G: ISMOC-4 h group.

Results of HE staining of AAR are shown in Figure 3(a). Massive necroses were found in the myocardial tissues of the Model-2 h and Model-4 h groups. The necroses were reduced in the HZOL-2 h, HZOL-4 h, ISMOC-2 h, and ISMOC-4 h groups, but the effects were more significant in the HZOL-4 h and ISMOC-4 h groups. TEM images of ultrathin sections of myocardial tissues are shown in Figure 3(b)A. We observed cardiomyocytes in a well-arranged myofilament and intercalated disc manner, as well as abundant normal mitochondria with no swelling, normal matrix density, and intact cristae, in the sham group (Figure 3(b)). However, in the I/R 2 h and I/R 4 h groups, myocardial I/R produced remarkable ultrastructural damages associated with irregularities and edematous separation of myofilaments and shortening of sarcomeres. Large areas of cytoplasmic vacuolization and mitochondrial swelling were evident with decreasing matrix density and cristae distortion (Figures 3(b)B and 3(b)C). HZOL treatment showed clear protection with relatively parallel arrangement of myofilaments and normal sarcomeres. Mitochondria were normal with mild swelling, normal matrix density, and slightly damaged cristae. However, mild cytoplasmic rarefaction with mild edema could still be observed (Figures 3(b)D and 3(b)E). These results were consistent with those of ISMOC (Figures 3(b)F and 3(b)G).

### 3.4. Effects of HZOL Pretreatment for I/R 2 h and I/R 4 h on Myocardial Apoptosis and Caspase-3 Expression

**Figure 4:**
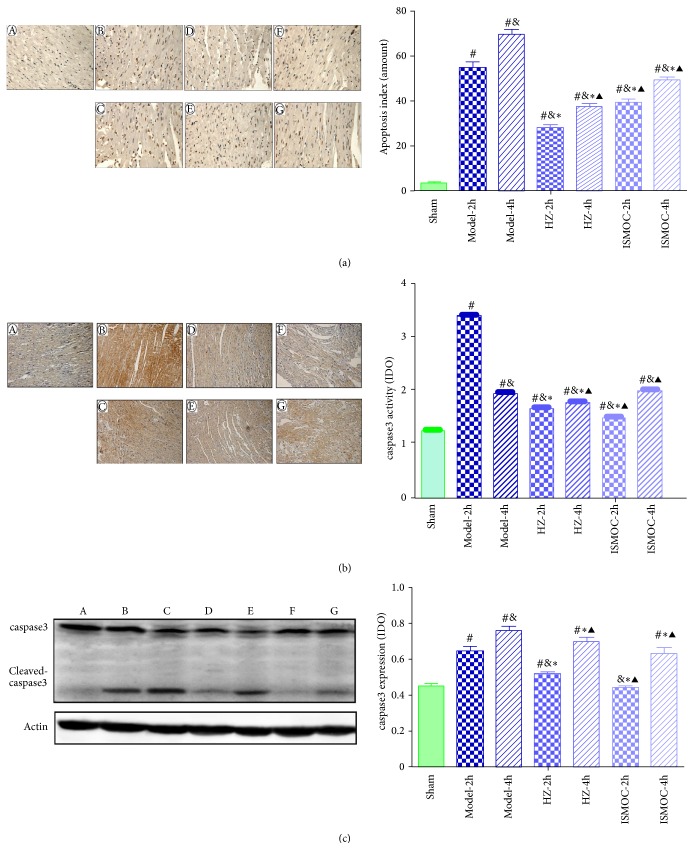
Effects of HZOL on apoptosis and expression of caspase-3. (a) TUNEL analysis of apoptosis in left ventricular AAR. (b) Immunohistochemical assay of caspase-3 expression. (c) Western blotting assay of caspase-3 expression. A: sham group; B: Model-2 h group; C: Model-4 h group; D: HZOL-2 h group; E: HZOL-4 h group; F: ISMOC-2 h group; G: ISMOC-4 h group; ^#^ *P* < 0.05 versus sham group; ^&^ *P* < 0.05 versus Model-2 h group; ^∗^ *P* < 0.05 versus Model-4 h group; ^▲^ *P* < 0.05 versus HZOL-2 h group.

TUNEL staining suggested that more brown stained cells were found in the Model-2 h and Model-4 h groups than those in the sham group (*P* < 0.05). Compared with the Model-2 h and Model-4 h groups, HZOL significantly decreased the number of apoptotic cells (*P* < 0.05) (Figure 4(a)).

Western blot showed that caspase-3 expression (*P* < 0.05) (Figure 4(c)) in the Model-2 h and Model-4 h groups significantly increased than that in the sham group. Compared with the Model-2 h and Model-4 h groups, the HZOL-2 h, HZOL-4 h, ISMOC-2 h, and ISMOC-4 h groups significantly decreased caspase-3 expression (*P* < 0.05). The effects were more significant in the HZOL-4 h and ISMOC-4 h groups. Compared with the HZOL-4 h group, the HZOL-2 h group significantly decreased caspase-3 expression (*P* < 0.05). The immunohistochemical results were similar to those of western blot (Figure 4(b)).

### 3.5. Effects of HZOL Pretreatment for Expression Levels of p53, Fas, and Bcl-2/Bax in I/R 2 h and I/R 4 h

**Figure 5:**
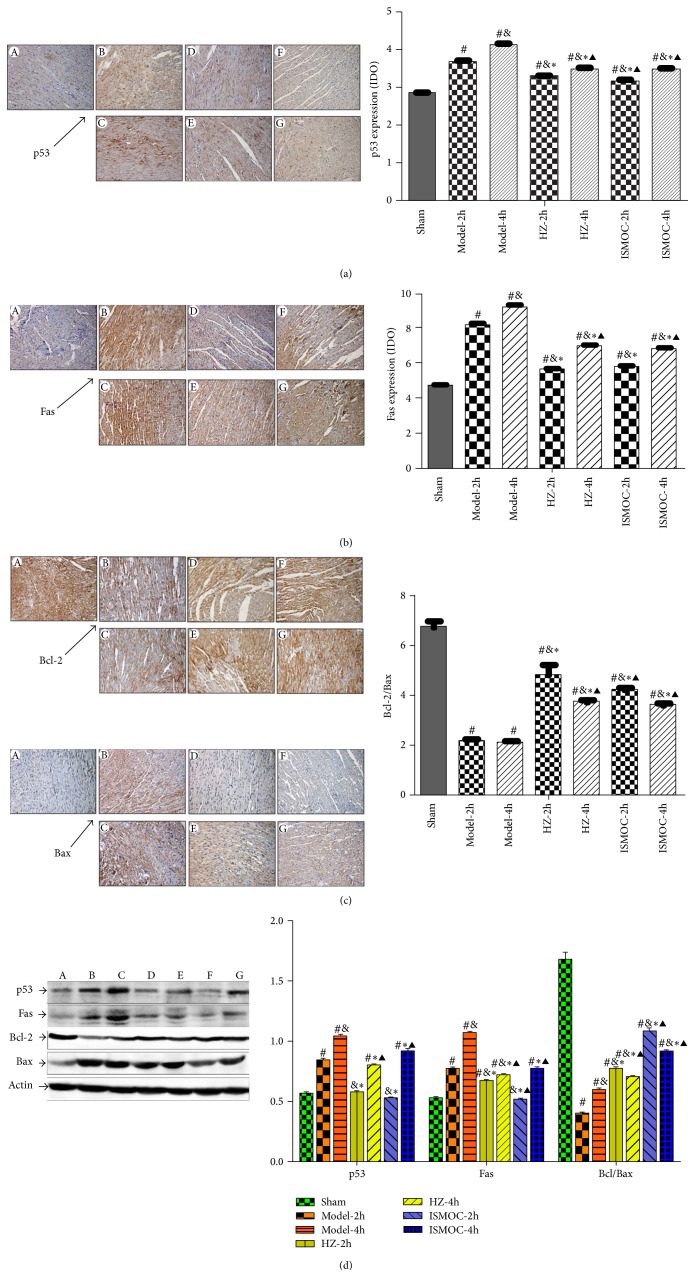
Effects of HZOL on apoptosis proteins expression. (a) Immunohistochemical assay of p53 expression. (b) Immunohistochemical assay of Fas expression. (c) Immunohistochemical assay of Bcl-2 and Bax expression. (d) Western blotting assay of p53, Fas, Bcl-2, and Bax expression. A: sham group; B: Model-2 h group; C: Model-4 h group; D: HZOL-2 h group; E: HZOL-4 h group; F: ISMOC-2 h group; G: ISMOC-4 h group; ^#^ *P* < 0.05 versus sham group; ^&^ *P* < 0.05 versus Model-2 h group; ^∗^ *P* < 0.05 versus Model-4 h group; ^▲^ *P* < 0.05 versus HZOL-2 h group.

The expression levels of p53, fas, and Bcl-2/Bax are shown in Figure 5. The immunohistochemical results show that p53 and fas significantly increased, whereas Bcl-2/Bax significantly decreased in the Model-2 h and Model-4 h groups than those in the sham group (*P* < 0.01). However, the HZOL-2 h, ISMOC-2 h, HZOL-4 h, and ISMOC-4 h groups could improve myocardial apoptosis by decreasing p53 and fas and increasing Bcl-2/Bax expression (*P* < 0.01). The effects were more significant in the HZOL-4 h and ISMOC-4 h groups (*P* < 0.01). Compared with the HZOL-2 h group, the HZOL-4 h group was more significant in decreasing p53 and fas and increasing Bcl-2/Bax expression (*P* < 0.01) (Figures 5(a) to 5(c)). Western blot results were similar to those of immunohistochemical analysis (Figure 5(d)).

## 4. Discussion

Our results indicate that the activation of HZOL at the beginning of reperfusion produced a protective effect in I/R injury and participated in a cascade of events that resulted in apoptosis. This study is the first to demonstrate the protective effect of HZOL inhibition against apoptosis caused by I/R injury.

The effects of HZOL prevented arrhythmias in rats by myocardial I/R for 2 and 4 h. Naked eye observations showed that the color of the heart in the model group was paler than that in the sham group. Almost all rats in the Model-2 h and Model-4 h groups experienced obvious ST-segment elevation, VT occurrence, and high VF frequency, which was the frequency of arrhythmias. By contrast, neither VT nor VF was observed in the sham group, and only a few ventricular premature beats appeared during the entire procedure. The serum results illustrate that CK-MB, CTnI, CRP, and MDA significantly increased, whereas SOD significantly decreased in the Model-2 h and Model-4 h groups (*P* < 0.05). All these parameters indicate severe myocardial ischemia. However, the HZOL-2 h, ISMOC-2 h, HZOL-4 h, and ISMOC-4 h groups could improve cardiac function by decreasing CK-MB, CTnI, CRP, and MDA and increasing SOD levels (*P* < 0.01). These effects were more notable in the HZOL-4 h and ISMOC-4 h groups. CK-MB, CTnI, and CRP are important indicators of cardiac function. MDA is an unsaturated fatty acid in free radical and lipid peroxidation metabolites. The content of MDA, which is an indirect marker of cellular damage, reflects the extent of systemic lipid peroxidation. The antioxidant SOD protects cells by reducing free radical-induced injury. SOD levels reflect the body's capacity to scavenge oxygen free radicals [11–13]. In the present study, myocardial SOD activity was attenuated in the Model-2 h and Model-4 h groups, combined with increased MDA content in the Model-2 h and Model-4 h groups. Our data suggest that myocardial oxidative stress may exacerbate I/R injury. The HZOL-2 h and HZOL-4 h groups could improve cardiac function by decreasing MDA and increasing SOD levels (*P* < 0.01). HZOL prevented arrhythmias by improving both cardiac function and resistance to oxidative stress.

The use of HZOL has been practiced for many years in the Affiliated Hospital of Zhongshan University of Traditional Chinese Medicine and is highly effective in relieving hypertension and hyperlipidemia [5]. This TCM includes leech, rhubarb, and Fructus arctii in its recipe. Leech secretions include vasodilation, bacteriostatic, analgesic, anti-inflammatory, anticoagulant, and antiedematous effects, which eliminate microcirculatory disorders, restore the damaged vascular permeability of tissues and organs, eliminate hypoxia, reduce blood pressure, and increase immune system activity [14–16]. Rhubarb has antioxidant, antiplatelet, and anticoagulant activities and can treat experimental jaundice in rats [17, 18]. Arctiin is one of the major lignans in Fructus Arctii that can enhance immunological function and acts as an anti-inflammatory agent [19], platelet-activating factor antagonist [20], Ca^2+^ antagonist, and antihypertensive agent [21, 22]. Moreover, arctiin can be metabolized into arctigenin by human intestinal bacteria [23]. Thus, the direct protective effects of HZOL on ischemia-induced arrhythmias can be assumed. Our research is related to these active constituents, especially those associated with the antiapoptotic components of HZOL.

Apoptosis typically proceeds through one of two signaling cascades, known as extrinsic and intrinsic pathways, both of which converge to activate the executioner caspase-3 [24]. The results in Figure 4 show that caspase-3 expression significantly increased in the Model-2 h and Model-4 h groups than that in the sham group. The HZOL-2 h, HZOL-4 h, ISMOC-2 h, and ISMOC-4 h groups significantly decreased caspase-3 expression and the number of apoptotic cells (*P* < 0.05). The extrinsic pathway is initiated by binding of death receptors, such as fas or tumor necrosis factor (TNF), with their respective ligands (fas ligand and TNF-*α*). These proteins further bind initiator procaspase-8 (or procaspase-10) and form the death-inducing signaling complex (DISC), thereby enabling their autoactivation [25–27]. Depending on the efficiency of DISC formation, activated caspase-8 can either directly activate the downstream executioner caspase-3 [28] or initiate the cleavage of the proapoptotic BH3-interacting domain death agonist (Bid), which subsequently engages the mitochondrial apoptotic cascade [29, 30]. The intrinsic (mitochondrial) pathway is activated by stimuli that trigger the permeabilization of the outer mitochondrial membrane followed by the release of proapoptotic proteins from the mitochondrial intermembrane space, leading to executioner caspase activation [31, 32]. This pathway is regulated by members of the Bcl-2 family of proteins that contain one or more Bcl-2 homology (BH) domains [33–35]. By contrast, the proapoptotic proteins Bax and Bak contain BH domains 1 to 3 [36, 37]. A larger group of proapoptotic proteins, including Bcl-2-associated death promoter, Bcl-2-interacting mediator of cell death, and Bid, contains only the BH3 domain. The Bcl-2 and Bax protein levels are directly related to apoptosis regulation. The increase in Bax levels promotes cell apoptosis, whereas Bcl-2 increases the inhibition of cell apoptosis; the Bcl-2/Bax ratio determines the viability of cells after apoptotic stimulation [38, 39]. The activation of p53 triggers apoptosis by transcriptional activation of proapoptotic genes and transcription-independent mechanisms. p53 can mediate apoptosis by inducing the expression of Bax [40] and other proapoptotic proteins. p53 induced by transient ischemia possibly switches off Bcl-2 expression and switches on Bax expression within the discrete area [38]. In this context, apoptosis of myocardial I/R in myocardial cells may be commonly regulated by endogenous and exogenous apoptosis mechanisms, thereby initiating the apoptotic factor caspase-3, which results in apoptosis of myocardial cells. Results show that HZOL increased the Bcl-2/Bax ratio and decreased the activities of p53, fas, and caspase-3. HZOL reduced the apoptosis rate induced by ischemia through decreased caspase-3 expression. This result occurred in association with heart failure and apoptosis of experimental animals [41]. In TEM, a large area of cytoplasmic vacuolization and mitochondrial swelling was evident with decreasing matrix density and cristae distortion in the I/R 2 h and I/R 4 h groups. Results of HZOL treatment showed normal mitochondria with mild swelling, normal matrix density, and slightly damaged cristae. HZOL ameliorated myocardial I/R through multiple apoptosis-related signal pathways that decreased p53, fas, and caspase-3 and increased the Bcl-2/Bax ratio. The regulation of these factors on apoptosis will be instrumental in defining novel therapeutic approaches to ischemic injury.
